# Impact of Caloric Restriction in Breast Cancer Patients Treated with Neoadjuvant Chemotherapy: A Prospective Case Control Study

**DOI:** 10.3390/nu15214677

**Published:** 2023-11-04

**Authors:** Isabella Castellano, Francesco Gallo, Paola Durelli, Taira Monge, Maurizio Fadda, Jasna Metovic, Paola Cassoni, Fulvio Borella, Carlo Raucci, Monica Menischetti, Alessandra Beano, Giuseppe Migliaretti, Concetta Finocchiaro

**Affiliations:** 1 Pathology Unit, Department of Medical Sciences, City of Health and Science University Hospital, University of Turin, 10126 Turin, Italy; isabella.castellano@unito.it (I.C.); paola.cassoni@unito.it (P.C.); 2 Dietetic and Clinical Nutrition Unit, City of Health and Science University Hospital, 10126 Turin, Italy; 3 Gynecology and Obstetrics 1U, Department of Surgical Sciences, City of Health and Science University Hospital, University of Turin, 10126 Turin, Italy; fulvio.borella@unito.it; 4 Oncology Unity, Cottolengo Hospital, 10152 Turin, Italy; 5 Medical Breast Oncology Unit, Department of Oncology, City of Health and Science University Hospital, University of Turin, 10126 Turin, Italy; 6 Department of Public Health and Pediatric Sciences, University of Turin, 10126 Turin, Italy

**Keywords:** breast cancer, neoadjuvant chemotherapy, caloric restriction diet, body composition, diet

## Abstract

**Background and aims:** It is well established that caloric restriction (CR) may influence metabolic and hormonal factors involved in cancer development and progression. Recently, several studies have demonstrated that CR may have a favorable impact on the response to systemic therapy in breast cancer (BC) patients. However, there is a lack of data regarding the influence of CR during neoadjuvant chemotherapy (NACT). Our study’s primary aim was to evaluate CR’s impact on BC patients undergoing NACT. Secondly, we investigated the nutritional efficacy and safety of this intervention. **Methods**: We performed a prospective, case–control study in two breast units. A diet group consisting of 39 patients undergoing NACT and CR was enrolled in our study at the same time. CR consisted of a 30% reduction in caloric intake, which increased to 50% on the days before, during, and after the administration of chemotherapy. A control group of 60 patients that underwent the same treatment approach only followed the general dietary recommendations for BC according to WCRF guidelines. The diet group was monitored during the study for both dietary adequacy and weight trends. **Results:** CR combined with NACT showed a statistically significant therapeutic response in tumor size (OR 2.94, IC 1.07–8.01, *p* = 0.009) and lymph node status (OR 3.22, IC 1.22–8.56, *p* = 0.001) compared to NACT alone, even after the adjustment for all biological parameters. Our data also showed the efficacy and safety of this intervention in both anthropometric and biochemical analyses. **Conclusions:** Patients who adhered to CR showed a better response to NACT, both in the breast and in the axillary lymph nodes, compared to the patients in the control group. Furthermore, the CR diet combined with NACT showed good tolerance and safety.

## 1. Introduction

Breast cancer (BC) is the most common cancer and the fifth leading cause of cancer deaths worldwide [1]. BC accounts for about 24.5% of cancer cases and 15.5% of cancer deaths, ranking first in incidence and mortality in most countries [1].

Due to early detection and improved therapies, BC survival rates have generally increased [2,3,4]; however, several genetic and epigenetic factors still have a significant prognostic role.

BC risk and progression are associated with overweight and obesity (Body Mass Index—BMI greater than or equal to 25 kg/m^2^) through a chronic inflammatory stimulus caused by dysfunctional adipocytes that increase circulating levels of cyclooxygenase 2, tumor necrosis factor α, interleukin 1β, and aromatase [5,6]. Moreover, obesity combined with high blood pressure, insulin resistance, and dyslipidemia results in a complex condition called metabolic syndrome. According to a recent meta-analysis, patients with metabolic syndrome have a statistically significant relative risk (RR: 1.47) of BC development compared to the healthy population (RR: 1.48, 95% Confidence Interval—CI 1.15–1.87, *p* < 0.002), especially for post-menopausal women [7]. Several preclinical studies have shown that caloric restriction (CR), defined as a 30% reduction in normal food intake without malnutrition, may interfere with cancer cell metabolism through three main mechanisms: substrate deprivation, regulation of oncogenes and tumor suppressor genes, and anti-inflammatory action. The link between CR and cancer biology stems from autophagy, essential for maintaining cellular balance and survival in response to normal and abnormal conditions. Autophagy breaks down and recycles cells through the lysosomal system; it operates at a steady, basal level but can be triggered by various factors, such as chemotherapy, hypoxia, DNA damage, and nutrient deficiencies [8]. Recently, CR has also been associated with increased efficacy of conventional therapies, such as chemotherapy (CT) and radiotherapy [9,10,11,12,13]. However, very few studies are available regarding CR influence during neoadjuvant CT (NACT) in BC patients [14]. 

Given these premises, the primary aim of our study was to compare the impact of CR on BC patients who underwent NACT. Secondly, we evaluated the nutritional efficacy and safety of this intervention.

## 2. Material and Methods

### 2.1. Study Design and Patients

We conducted a prospective study of patients who received NACT treatment for BC between February 2018 and December 2021 at two breast units (Città della Salute e della Scienza Hospital and Cottolengo Hospital Turin, Italy).

The eligibility criteria were (i) age between 18 and 70 years old, (ii) no previous treatment for other neoplasia, (iii) performance status ECOG < 2, and (iv) BMI between ≥18 kg/m^2^ and ≤35 kg/m^2^ (without weight decrease of > 5% in the last 3 months). The exclusion criteria were (i) being underweight (BMI < 18 kg/m^2^), (ii) history of eating disorder, (iii) renal failure, (iv) type 2 diabetes mellitus, and (v) psychiatric co-morbidity.

Before any treatments (T0), in all cases, tumor size was determined using magnetic resonance in addition to mammography. Data regarding histotype, grade, estrogen receptor (ER), progesterone receptor (PR), HER2, and Ki67, assessed on core biopsy, were collected. Axillary status was defined using ultrasound and, in case of doubt, confirmed with fine-needle aspiration and/or core needle biopsy. All patients received a standard NACT schedule: epirubicin (90 mg/m^2^) and cyclophosphamide (600 mg/m^2^) every 3 weeks for 4 cycles (T1), followed by weekly paclitaxel (80 mg/m^2^) for 12 weeks (T2). In the case of HER2-positive BC, trastuzumab was also administered. At the end of treatment (T2), patients underwent magnetic resonance and axillary ultrasound to evaluate the radiological response and determine the correct surgical approach. After NACT, the patients underwent surgery, and the pathological response was evaluated on surgical specimens. Pathological complete response (pCR) was defined as the absence of an invasive tumor, except for the presence of intraductal carcinoma (ypT0/Tis, ypN0). If present, the residual tumor was classified according to both the TNM staging system and Pinder classification [15], according to our national guidelines (Gipam-Siapec) [16]. Moreover, the tumor size, ER, PR, HER2, Ki67 expression, and lymph node status were assessed. 

**Figure 1:**
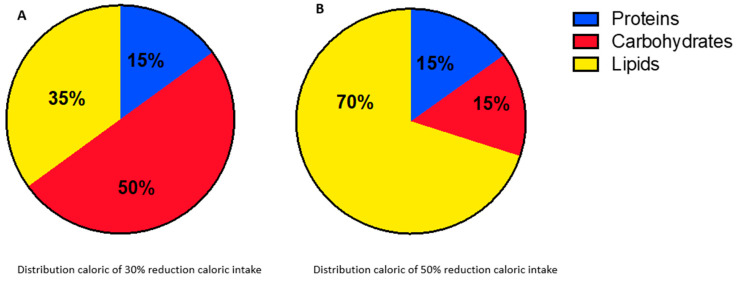
(**A**,**B**) Different caloric intake distributions in the two moments of caloric restriction.

During NACT, patients maintained a diet with a 30% reduction in caloric intake (70% of total energy expenditure, TEE) composed of 15% proteins, 50% carbohydrates (CHO), and 35% lipids (Figure 1A). In addition, in the 24 h preceding CT, on the day of CT, and the day after CT, the calorie intake was reduced to 50% compared to the total energy requirement at the initial time and was composed of 15% proteins, 15% CHO, and 70% lipids (Figure 1B).

The patients who underwent a reduction in BMI to <18 kg/m^2^ were hospitalized due to CT side effects, experienced a weight decrease of >3% in 3 months or >5% in 6 months (compared to their ideal weight), and/or withdrew their consent were excluded from the study.

**Figure 2:**
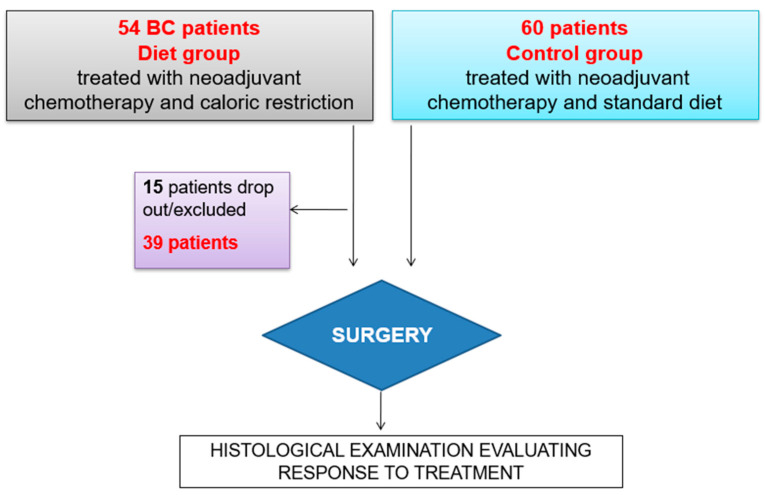
Two groups of patients under treatment with caloric restriction and standard diet.

The response to NACT was evaluated by comparing the cases of those who strictly followed the CR plan (diet group) with a series of BC patients who underwent NACT in the same period (control group) who followed dietary recommendations for BC according to the World Cancer Research Fund guidelines (Figure 2).

### 2.2. Nutritional Assessment and Blood Sampling

The CR group was closely monitored during NACT to evaluate the effectiveness of anthropometric and nutritional parameters and, therefore, safety and feasibility. We assessed body weight, BMI, and waist and hip circumferences monthly. More precisely, the weight was measured using a mechanical column scale (SECA 700, SECA GMBH & Co. KG, Hamburg, Germanu) with a maximum capacity of 200 kg and with an altimeter at 220 cm; the weight was measured with a sensitivity of 0.1 kg and the height with a sensitivity of 0.5 cm. The patient was weighed without shoes and with light clothing. Body circumferences were measured using a flexible and inelastic anthropometric meter equipped with a centimeter scale.

Furthermore, food intake was evaluated monthly through a qualitative and quantitative “24-h-recall” method. The food history was taken by a registered dietitian who assessed diet compliance regarding proteins, lipids, carbohydrates, and caloric intake. Compliance with the CR diet scheme was evaluated by considering women who did not exceed by more than 15% the total calories prescribed daily, both in the 50% and 70% diet schemes. 

Moreover, body composition was assessed via tetrapolar single-frequency bioimpedentiometry (BIA) (STA-BIA 50 kHz, Akern, Florence, Italy) and indirect calorimetry (IC) using the Deltatrac II (DATEX, Division of Instruments Corp., Helsinki, Finland) at T0 and T2. As shown in other studies, these tools are useful to monitor body composition parameters (such as phase angle—PA, total body water—TBW, fat-free mass—FFM, fat mass—FM, and body cell mass—BCM) and variations in resting energy expenditure (REE) [17]. The procedures were applied following ESPEN Working Group Protocols [18]. Respiratory quotient (RQ) and REE were measured with IC. RQ was used to detect the rate of energy expenditure by determining the inspired and expired concentrations of oxygen (VO2) and carbon dioxide (VCO2), reflecting nutrient metabolism.

Blood samples from the CR group were collected at T0, T1, and T2 for nutritional evaluation. The effects of CR were determined by recording metabolic parameters (insulin, glucose, and homeostatic model assessment—HOMA), endocrine parameters (such as thyroid-stimulating hormone and triiodothyronine), hematologic parameters (erythrocyte, thrombocyte, and leukocyte count), and inflammatory response (C-reactive protein). Also, other markers were analyzed to monitor the overall status of patients (creatinine, aspartate aminotransferase, alanine aminotransferase, gamma-glutamyl transferase, alkaline phosphatase, total cholesterol, high-density lipoprotein (HDL), low-density lipoprotein (LDL), triglycerides, uric acid, total protein, albumin, and transferrin). The routine blood chemistry exams were conducted before each CT administration.

### 2.3. Statistical Analysis

We use descriptive statistical indicators (mean with standard deviation and percentiles for continuous variables and percentages for categorical ones) to describe the case studies and to identify any confounding factors [19,20].

We used the χ^2^ test to evaluate any differences between the diet group and control group at T0 and T2 for categorical measures. We used the McNemar test to evaluate the change between T0 and T2 of lymph node status [19,21].

To assess the effects of CR on NACT response, we performed logistic regression models. We considered age and tumor size at T0 as confounding factors. We further adjusted the association for tumor size, Ki67, ER, HER, and PGR measured at T0. We reported the results as crude and adjusted odd ratios (OR) and relative 95% CI. We used SAS® 9.4 software (SAS Institute, Inc., Cary, North Carolina) for statistical analysis.

## 3. Results

### 3.1. Impact of CR in Response to NACT

A total of 54 patients were enrolled in the diet group along with 60 patients belonging to the control group (Figure 2). A total of 15 patients belonging diet group voluntarily dropped out of the study, while 39 completed the duration of CR.

The clinical and pathological characteristics before starting NACT are reported in Table 1. At T0, the median age was 46 and 52 for the diet and control groups, respectively. Tumor size, as revealed via magnetic resonance, was significantly larger in the control group, compared to the diet group (*p =* 0.003). Additionally, BC in the diet group showed ER and PR positive expression less frequently (*p* = 0.01 and *p* = 0.02, respectively). We did not observe significant differences regarding other parameters (Table 1).

As shown in Table 2, control-group patients were more frequently treated with mastectomy (36/60), while only seven diet-group patients (7/39) underwent the same surgical approach (*p* = 0.008). Even after NACT, the diet group in general demonstrated smaller tumors compared to the control group (*p* = 0.009) and more frequently showed residual tumors (*p* = 0.015).

After NACT, control-group patients were in general associated with higher stages in terms of ypT (*p =* 0.001) and ypN status (*p* = 0.008) (Table 2).

No BC in the diet group was assigned to the Pinder 3 score (no signs of tumor regression), unlike 11/60 of the control group (*p* = 0.004).

Moreover, regarding lymph node assessment, 14/60 control-group patients had a Pinder 4 score (presence of metastasis and no signs of tumor regression), while the majority of CR cases had Pinder 1 and Pinder 2 scores (30/39) (*p* = 0.001) (no presence of metastasis) (Table 2).

As shown in Table 3, in the CR group, we observed that only nine patients remained positive after NACT, while 14/23 (60.8%) were downstaged to yPT0. Otherwise, in the control group, only 16/45 (35.5%) patients did not have positive lymph nodes after NACT treatment (McNemar test: χ^2^ = 3.96, *p* = 0.042).

Logistic regression was conducted to evaluate the association between CR and response to NACT, considering both tumor and lymph node status.

As shown in Table 4, we demonstrated a positive and statistically significant association between CR and achievement of pCR for both BC and lymph nodal parameters (crude ORs 2.66 95% CI [1.15–6.18] and 4.05 95% CI [1.72–9.52], respectively). The association remains fully significant for both BC and lymph nodes even after the adjustment for tumor size and for ER, PR, Ki67, and HER2 expression measured at T0: adjusted ORs 2.94 95% CI [1.07–8.10] and 3.22 95% CI [1.22–8.56], respectively.

### 3.2. Nutritional Efficacy and Safety of CR in NACT Patients

#### 3.2.1. IC and BIA

IC was performed at T0 and T2 in the diet group. We found no statistically significant variation in RQ obtained with IC (*p =* 0.16). However, we observed a statistically significant variation in REE from the beginning to the end of our study period (*p* = 0.00069).

At T0 and T2, BIA was also employed. We found a statistically significant reduction in phase angle (PA, *p =* 0.003) and reactance (Xc, *p* = 0.00093), and we detected a noticeable reduction in fat mass (FM, *p* < 0.0001). As for BCM, there was a difference at the threshold of statistical significance (*p =* 0.023). On the other hand, we did not observe any significant change in resistance (Rz, *p =* 0.42). Concerning water distribution, we found no significant variations (TBW, *p* = 0.55) (Table 5).

#### 3.2.2. Anthropometric Parameters

Anthropometric parameters at T0 and T2 of the diet group are shown in Table 6.

We noted that there was a significant reduction in weight between T0 and T1 (*p* < 0.0001) and between T0 and T2 (*p* < 0.0001), but no significant difference was observed between T1 and T2 (*p* = 0.9958). About the weight trend, we recorded a significant reduction in BMI between the start and the end of the study period (*p* = 0.0204), but no statistically significant difference was noted between T1 and T2 (*p* = 0.9951). Regarding circumferences, we noted that there was a statistically significant reduction in waist circumference in all intervals of time taken into account in our study, especially during the first three months (*p* < 0.0001). A similar trend was observed in the data concerning hip circumferences, with a statistically significant reduction (*p* < 0.0001).

#### 3.2.3. Biochemical Analysis

We also investigated the blood nutritional chemistry and inflammatory state of the patients from T0 to T2, but we did not find anything that would affect tolerance to CR.

Regarding protein synthesis parameters, we observed no statistically significant difference in the three time intervals considered for total protein. Blood albumin showed a statistically significant difference between T0 and T2 (*p* = 0.0296) and T1 and T2 (*p* = 0.0006) but not between T0 and T1 (*p* = 0.1244). The transferrin trend showed a significant difference only between T0 and T2 (*p* = 0.0444).

As for the glycemic profile of the diet group, we found no difference in blood glucose. Instead, blood insulin and HOMA index showed a statistically significant difference and reduction in the T0–T1 and T0–T2 periods, but not in the T1–T2 period (insulin: T0–T1, *p* = 0.0004; T0–T2, *p* = 0.0001; HOMA index: T0–T1, *p* = 0.0052; and T0–T2, *p* = 0.0025).

We analyzed total LDL and HDL cholesterol, reaching statistical significance in all intervals of time only with LDL cholesterol (T0–T1, *p* < 0.0001; T1–T2, *p* = 0.0032; T0–T2, *p* < 0.0001), with contrasting results on total and HDL cholesterol.

We also investigated the blood C-reactive protein and protein synthesis parameters; we did not detect any statistically significant difference (Supplementary Tables S1 and S2).

#### 3.2.4. Compliance with CR

Compliance with the CR diet was evaluated by considering compliant women who did not exceed more than 15% of the total calories prescribed daily, both in the 30% and 50% diet schemes. Energy and macronutrient intakes were reported in Table 7.

The diet group showed a median compliance of 80% in both diet schemes. We observed a decrease in compliance in both diets during the third and fourth months of the dietetic program, mainly due to the increase in CT side effects or the beginning of new side effects.

More specifically, we observed a higher calorie intake in the 50% diet scheme (a median value of 980 kcal/per day compared to the 900 Kcal/per day prescribed during the third month) and a higher intake in CHO during the third month, especially in the more restricted days (in the 50% diet, a median value of 60 g/per day compared to the 47 g/per day prescribed and in the 70% diet, a median value of 120 g/per day compared to the 117 g/per day prescribed), mainly due to the increased consumption of so-called “comfort food” (more fresh fruits, cookies, and chocolate), as reported by patients during the monthly nutritional assessment. The monthly nutritional assessment allowed us to give the BC group a constant reminder of the objectives of the CR, and it contributed to enhancing the compliance of the patients.

## 4. Discussion

The role of periodic fasting in cancer prevention has been widely studied [22,23,24,25,26]. However, to date, little is known about the impact of CR during BC therapy. In particular, although some researchers have reported that weight loss may negatively affect the prognosis of cancer patients, short-term fasting and CR in basic and clinical settings have shown their efficacy in increasing response to CT [27].

In line with these results, we performed an observational study, recruiting BC patients who underwent both NACT and CR.

The results of our study suggest that CR has the potential to enhance the effect of NACT, showing good tolerance and safety.

Specifically, we observed that patients who followed CR achieved pCR more frequently than patients following a standard diet. This positive effect remained significant after adjustment for the size of the lesion and other BC parameters strongly related to the NACT response, such as ER, PR, HER2, and Ki67.

Additionally, for the first time, our study showed that CR may have a specific role in axillary lymph node status: we observed that 14/23 (60.8%) of the diet-group patients were downstage to yPN0 after NACT. On the other hand, in the control group, 16/45 (35.5%) patients had no positive lymph nodes after NACT.

To the best of our knowledge, only a few studies have analyzed a CR during CT treatment for BC [28,29,30,31] and only one proposed this approach during NACT. Specifically, De Groot et al. randomized 131 BC patients to receive either a fasting-mimicking diet or their regular diet for 3 days before and during NACT. Their results showed that a radiologically complete response occurred more often in patients using the fasting-mimicking diet, and they also confirmed that this approach was safe and effective as an adjunct to CT in BC patients [32].

Several experimental models have shown that fasting may improve CT response through different molecular mechanisms [28], for example, by decreasing the concentration of circulating glucose, insulin, and insulin-like growth factor-1 (IGF-1). All of these markers are involved in the activation of the PI3K/AKT/mTORC1 axis, one of the most important proliferative pathways of cancer cells [33,34].

Experimental data show that the combination of short-term fasting cycles with CT is effective in enhancing tolerability and efficacy [27]. A series of studies in animal models have shown that periodic fasting can protect normal cells from the toxic effects of CT [35,36,37] and that CR can effectively delay the progression of several tumor types. Emerging data suggest that the CR approach may be beneficial in well-nourished patients undergoing prolonged intensive and toxic oncologic therapy [38].

BC is correlated with insulin resistance and high insulin levels; one proposed mechanism by which CR inhibits tumor growth is its modulation of circulating levels of free IGF-1 in the serum [22,39,40], responsible for malignant transformation and neo-angiogenesis.

In women who fasted for 24 h before NACT, decreased hematological toxicity and DNA damage in circulating mononuclear cells were reported. This decreased cytotoxic effect of fasting may be explained by the reduced expression of several oncogenes in healthy cells, such as RAS and the AKT signaling pathway, as well as a drop in circulating insulin, IGF-1, glucose adiponectin, leptin, VEFG, PAI-1, TNFa, IL-6, and MCP1, while levels of IGFBP1 and ketones in the body increase [38,41,42]. Based on these findings and our results, we can hypothesize the role of insulin in improving response to NACT among patients who adhered to CR.

Our results have shown that CR during CT resulted in a significant weight reduction both between T0 and T1 (*p* = 0.0001) and T0 and T2 (*p* = 0.0001), which stabilized over the last 3 months. This weight reduction may justify the PA, FFM, and REE decreases that we observed through the energy expenditure and body composition examinations.

We know that CT is associated with a loss of lean mass and an increase in fat mass, independent of weight change [43]. At the same time, the reduction in lean mass is strongly associated with the important decrease in FM in our study (*p* = 0.0001).

The results of our study showed that the CR protocol is safe because BCM, despite its slight reduction, remained within the normal range (*p* = 0.023).

The diet group showed a median compliance of 80% in both diets. We observed a decrease in compliance during the third and fourth months of the dietetic program, mainly due to the increase in NACT side effects or the onset of new side effects.

Given the adequate compliance of the patients with the proposed CR scheme and the stability of the blood chemistry parameters referred to as protein synthesis, this diet scheme can be considered safe.

As for protein synthesis parameters, total protein had a small reduction, as did blood albumin. Only transferrin, in its mean values, showed a significant rise between the beginning and the end of our protocol.

## 5. Conclusions

The results of our study demonstrated that CR in BC patients treated with NACT may have a favorable impact in terms of breast pCR and downstaging axillary lymph nodes.

Indeed, the use of CR diets in patients with BC still needs to be confirmed by more data from the international literature, but soon, it could be a new additional therapeutic tool to be used simultaneously with traditional therapies on selected groups of patients with certain characteristics and by expert staff. Moreover, considering the absence of significant side effects developed by the patients, our study confirmed the nutritional efficacy and safety of CR during CT and opened the way to extend this approach to other contexts of oncological care. Further studies will be needed to optimize its use.

## Figures and Tables

**Table 1:** Clinic–pathological characteristics of patients who underwent caloric restriction protocol compared to free-diet patients before starting any treatment (T0).

	Diet Group (39)	Control Group (60)	Chi-Square	*p*-Value
**Age years (median)**	46	52		
**Size (mm)**				
≤20	11	3	11.586	0.003
20–50	21	36		
>50	7	21		
**Lymph node tatus**				
Positive	23	45	2.822	ns
Negative	16	15		
**Histotype**				
Non-special type	39	55	3.423	ns
Others	0	5		
**Histological grade**				
1	2	1	1.22	ns
2	17	24		
3	20	35		
**ER (%)**				
0	17	12	6.35	0.01
>1	22	48		
**PR (%)**				
0	26	26	5.161	0.02
>1	13	34		
**HER2**				
Negative	21	33	0.013	ns
Positive	18	27		
**Ki67 (%)**				
≤20	2	8	1.752	ns
>20	37	52		

ER: estrogen receptor, PR: progesteron receptor, and ns: not significant.

**Table 2:** Clinic–pathological characteristics of patients underwent caloric restriction protocol compared to control-group patients after neoadjuvant treatment (T2).

	Diet Group (39)	Control Group (60)	Chi-Square	*p*-Value
**Surgery**				
Conservative	32	24	6.915	0.008
Mastectomy	7	36		
**Size (mm)**				
Not determined	11	7	11.505	0.009
≤20	19	23		
20–50	9	20		
>50	0	10		
**Residual tumor**				
No	20	17		0.015
Yes	19	43		
**Lymph node status**				
Positive	9	29	6.375	0.01
Negative	30	31		
**ypT**				
Is	1	7	22.324	<0.001
0	19	10		
1	17	24		
2	1	13		
3	0	6		
4	1	0		
**ypN**				
0	30	29	9.431	0.008
1	7	29		
2	2	2		
**Pinder tumor stage**				
1	20	17	10.767	0.004
2	18	32		
3	0	11		
**Pinder lymph node stage**				
1	24	17	15.081	0.001
2	6	14		
3	9	17		
4	0	12		
**ER (%)**				
0	9	8	5.478	0.01
>1	10	35		
**PR (%)**				
0	14	22	4.938	0.02
>1	3	21		
Not performed	2	0		
**HER2**				
Negative	13	33	0.477	ns
Positive	6	10		
**Ki67 (%)**				
≤20	9	25	0.166	ns
>20	5	18		
Not performed	5	0		

ER: estrogen receptor, PR: progesteron receptor, and ns: not significant.

**Table 3:** Changes in lymph node status after NACT treatment in diet and control groups.

			Lymph Nodes Status Post Nact		Total
			yN0	yN1–2	
Control Group	Lymph Nodes Status Pre Nact	Negative	13	2	15
		Positive	16	29	45
		Total	29	31	60
Diet Group		Negative	16	0	16
		Positive	14	9	23
		Total	30	9	39

McNemar test: χ^2^ = 3.96, *p* = 0.042. NACT: neoadjuvant chemotherapy.

**Table 4:** Logistic regression model regarding pathological complete response vs. residual cancer in diet group compared to control group, in bold, crude OR.

pCR vs. Residual Cancer						
	Breast Cancer			Lymph Nodes		
	OR (Crude)	95% Confidence Interval		OR (Crude)	95% Confidence Interval	
		Inf	Sup		Inf	Sup
**Diet group vs. control group**	**2.66**	**1.15**	**6.18**	**4.05**	**1.72**	**9.52**
	**OR** **(adj)**	**Inf**	**Sup**	**OR** **(adj)**	**Inf**	**Sup**
Diet group vs. control group	2.94	1.07	8.10	3.22	1.22	8.56
Size in mm (T0)	0.94	0.44	2.01	0.77	0.36	1.63
ER (T0)	1.09	0.32	3.69	0.54	0.16	1.83
PGR (T0)	0.45	0.14	1.42	0.38	0.12	1.19
Ki67 (T0)	1.02	0.84	15.34	5.17	0.47	56.30
HER2 (T0)	1.66	1.05	2.63	1.09	0.69	1.73

ER: estrogen receptor, OR: odd ratio, pCR: pathological complete response.

**Table 5:** Body composition of 39 diet-group patients.

Parameter	Time	Median (Range)	*p*
**Resistance (Ohm)**	T0	**598** (416–700)	
	T2	**565** (426–727)	ns
**Reactance (Ohm)**	T0	**57** (40–75)	
	T2	**50** (37–66)	**0.0009**
**PA (°)** Reference values: 5.2–7.2°	T0	**5.6** (4.3–6.6)	
	T2	**5.0** (4.2–6.5)	**0.003**
**FFM (kg)**	T0	**44.2** (36.2–53.5)	
	T2	**45** (38.6–55.2)	ns
**FM (kg)**	T0	**22.4** (17.1–29.6)	
	T2	**12.1** (2.3–32)	**0.0001**
**TBW (L)**	T0	**32.4** (26.5–39.2)	
	T2	**32.5** (25.7–38.6)	ns

FM: fat mass, FFM: free fat mass, ns: not significant, PA: phase angle, and TBW: total body water.

**Table 6:** Anthropometric parameters of 39 diet-group patients from T0 to T2.

Median	T0	T2	*p*	Δ% T0–T2
**Body Weight (kg)**	63	59	0.001	−6.35
**BMI (kg/m^2^)**	24.22	22.49	0.02	−7.14
**Waist Circumference (cm)**	81	74	0.0001	−9.46
**Hip Circumference (cm)**	97.5	91.5	0.0001	−6.56

BMI: body mass index.

**Table 7:** Energy/macronutrient intake compared in 39 diet-group patients.

	Time	CR Diet 50% TEE		CR Diet 70% TEE
		Prescription	Value	Prescription
**Energy** (kcal/die) Median (range)	**T0**	**900** (704–1097)	**918** (450–1160)	**1300** (1012–1660)
	**T1**		**980** (532–1400)	
	**T2**		**945** (550–1126)	
**CHO** (g/die) Median (range)	**T0**	**47** (37–53)	**56** (20–117)	**117** (85–145)
	**T1**		**60** (20–126)	
	**T2**		**58** (30–110)	
**Protein** (g/die) **Median** (range)	**T0**	**75** (45–98)	**68** (31–83)	**65** (55–80)
	**T1**		**67** (34–100)	
	**T2**		**67** (33–82)	
**Lipid** (g/die) **Median** (range)	**T0**	**50** (37–59)	**49** (20–77)	**65** (44–92)
	**T1**		**52** (30–80)	
	**T2**		**50** (25–75)	

CHO: carbohydrate, CR: caloric restriction, and TEE: total energy expenditure.

## Data Availability

The data that support the findings of this study are available from the corresponding author on reasonable request.

## Supplementary Materials

The following supporting information can be downloaded at https://www.mdpi.com/article/10.3390/nu15214677/s1, Table S1: Protein synthesis parameters. Table S2: Blood concentration of C-reactive protein.

## Abbreviations

BC	Breast Cancer
BCM	Body Cell Mass
BIA	Bioimpedentiometry
BMI	Body Mass Index
CHO	Carbohydrates
CI	Confidence Intervals
CR	Caloric Restriction
CT	Chemotherapy
ER	Estrogen Receptor
FFM	Free Fat Mass
FM	Fat Mass
HDL	High-density Lipoprotein
HOMA	Homeostatic Model Assessment
IC	Indirect Calorimetry
LDL	Low-density Lipoprotein
NACT	Neoadjuvant Chemotherapy
OR	Odd Ratio
PA	Phase Angle
pCR	Pathological Complete Response
PR	Progesterone Receptor
REE	Resting Energy Expenditure
RQ	Respiratory Quotient
RR	Relative Risk
TEE	Total Energy Expenditure
TBW	Total Body Water
