# Supplementary material for: Impact of Caloric Restriction in Breast Cancer Patients Treated with Neoadjuvant Chemotherapy: A Prospective Case Control Study
Source: Nutrients. 2023 Nov 4;15(21):4677. doi: 10.3390/nu15214677 (PMC10648206; doi:10.3390/nu15214677)
Supplement: Supplementary file 1 [file nutrients-15-04677-s001.zip › nutrients-2662276-supplementary.pdf]

Supplementary Table S1: protein synthesis parameters

	TOT. PR. T0	TOT. PR. T1	TOT. PR. T2	ALBUMIN T0	ALBUMIN T1	ALBUMIN T2	TRANSFERRIN T0	TRANSFERRIN T1	TRANSFERRIN T2
N°	34	32	16	34	32	15	33	31	17
Missing	11	13	29	11	13	30	12	14	28
Mean	6.75	6.70	6.62	4.39	4.44	4.23	241	244	249
Median	6.80	6.65	6.70	4.50	4.50	4.20	242	226	238
Standard deviation	0.388	0.385	0.392	0.305	0.296	0.249	44.2	47.4	44.5
Minimum	5.90	5.90	5.70	3.80	3.90	3.90	168	170	184
Maximum	7.40	7.40	7.20	4.90	5.00	4.90	342	354	321

Supplementary Table S2: blood concentration of C-reactive protein

	CRP T0	CRP T1	CRP T2
N	33	31	15
Missing	12	14	30
Mean	5.41	2.83	1.81
Median	2.10	1.30	0.500
Standard deviation	7.03	3.77	2.45
Minimum	0.300	0.300	0.100
Maximum	29.1	17.6	7.60
